# Retraction: The role of thymosin beta 4 on odontogenic differentiation in human dental pulp cells

**DOI:** 10.1371/journal.pone.0320021

**Published:** 2025-03-03

**Authors:** The PLOS One Editors

After this article [1] was published, concerns were raised about Figs 1 and 3–7.

Specifically:

- When color levels are adjusted, there appear to be vertical discontinuities in multiple panels of Figs 1, 5 and 7.
- The following panels appear similar:
  ◦ In Fig 3B, lanes 4-6 of the GAPDH - OM panel and lanes 1–3 of the GAPDH – OM+Tβ4 panel.
  ◦ The GAPDH panels in Fig 4A (left column) and Fig 5C.
  ◦ Lanes 1-2 in the BMP4 panel in Fig 4A (left column) when flipped vertically, lanes 1–2 in the BMP2 panel in Fig 4A (left column) and lanes 1–2 in the BMP2 panel in Fig 7C.
  ◦ Lanes 1–3 in the BMP2 panel in Fig 4A (left column) and lanes 1–3 in the BMP2 panel in Fig 7C.
  ◦ Lanes 2–3 of the BMP2 panel in Fig 4A (right column) and lanes 2–3 of the BMP4 panel in Fig 7C.
  ◦ The β-actin – OM panels in Figs 4B and 6B.
  ◦ The β-actin – OM+Tβ4 panels in Figs 4B and 6B.
  ◦ Lanes 2–6 of the GAPDH panel in Fig 6A (right column) and the GAPDH panel in Fig 7C.

The authors did not respond to editorial requests for comment and underlying data. In light of the above unresolved concerns that question the integrity and reliability of the reported results and conclusions, the *PLOS One* Editors retract this article.

All authors either did not respond directly or could not be reached.
